# Examining the Differential Role of General and Specific Processing Speed in Predicting Mathematical Achievement in Junior High School

**DOI:** 10.3390/jintelligence10010001

**Published:** 2021-12-21

**Authors:** Dazhi Cheng, Kaihui Shi, Naiyi Wang, Xinyang Miao, Xinlin Zhou

**Affiliations:** 1 State Key Laboratory of Cognitive Neuroscience and Learning, IDG/McGovern Institute for Brain Research, Beijing Normal University, Beijing 100875, China; chengdazhi1015@126.com (D.C.); shikaihui0103@foxmail.com (K.S.); xmiao97@hotmail.com (X.M.); 2 Lab for Educational Neuroscience, Center for Educational Science and Technology, Faculty of Education, Beijing Normal University, Beijing 100875, China; wangnaiyi@bnu.edu.cn; 3 Advanced Innovation Center for Future Education, Beijing Normal University, Beijing 100875, China; 4 Department of Pediatric Neurology, Capital Institute of Pediatrics, Beijing 100020, China

**Keywords:** specific processing speed, general processing speed, mathematics, academic achievement, academic fluency

## Abstract

Processing speed is divided into general (including perceptual speed and decision speed) and specific processing speed (including reading fluency and arithmetic fluency). Despite several study findings reporting the association between processing speed and children’s mathematical achievement, it is still unclear whether general or specific processing speed differentially predicts mathematical achievement. The current study aimed to examine the role of general and specific processing speed in predicting mathematical achievements of junior high school students. Cognitive testing was performed in 212 junior school students at the beginning of the 7th grade year, along with assessment of general and specific processing speed. Relevant academic achievement scores were also recorded at the end of the 7th and 9th grade years. Hierarchical regression analyses showed that specific processing speed made a significant unique contribution in mathematical achievement by the end of the 7th grade and could significantly predict mathematical achievements in the high school entrance examinations by end of the 9th grade after controlling for age, gender, and general cognitive abilities. However, general processing speed could not predict mathematical achievements. Moreover, specific processing speed could significantly predict all academic achievements for both the 7th and 9th grade. These results demonstrated that specific processing speed, rather than general processing speed, was able to predict mathematical achievement and made a generalised contribution to all academic achievements in junior school. These findings suggest that specific processing speed could be a reflection of academic fluency and is therefore critical for long-term academic development.

## 1. Introduction

Processing speed refers to the cognitive ability to perform simple routine cognitive tasks quickly, regularly, and fluently. According to the Cattell–Horn–Carroll (CHC) theory of cognitive abilities, processing speed typically includes: perceptual speed, decision speed, arithmetic fluency, and reading fluency (Schneider and Mcgrew 2012). Several existing studies reported that processing speed is associated with children’s mathematical achievement (Bull and Johnston 1997; Cowan et al. 2011; Fuchs et al. 2006; Fung et al. 2014; Geary 2011; Hecht et al. 2001; Lambert and Spinath 2017; Leikin et al. 2014; Lin 2020; Passolunghi and Lanfranchi 2012; Rohde and Thompson 2007; Taub et al. 2008; Träff et al. 2017). 

Prior studies demonstrated that processing speed significantly predicted mathematical achievement. Specifically, symbolic processing speed is closely associated with children’s mathematical performance (Bull and Johnston 1997; Hecht et al. 2001; Lambert and Spinath 2017; Rohde and Thompson 2007). Rohde and Thompson (2007) reported that processing speed, which is assessed by symbolic visual matching—letters and numbers stimuli, constantly accounted for a significant amount of additional variance in mathematics performance. Additionally, processing speed could also predict mathematic achievement longitudinally (Cowan et al. 2011; Geary 2011; Passolunghi and Lanfranchi 2012; Träff et al. 2017). Path analyses models revealed the following findings about processing speed: (1) prediction power of number symbolic visual matching for numerical competence among pre-schoolers, and (2) prediction power for mathematical achievement among followed-up first graders (Passolunghi and Lanfranchi 2012). 

Furthermore, some studies have identified the role of arithmetic fluency in mathematical achievement. For example, Cowan et al. (2011) found that basic arithmetic fluency could predict mathematical achievement from the second to the third grade, and it also influenced mathematical problem solving, which is an aspect of mathematical achievement. Moreover, a recent meta-analysis indicated that arithmetic fluency is an essential predictor of word problem solving among older students (Lin 2020). For younger students, mathematics facts retrieval and mathematics computation were essential for word-problem solving, whereas older students relied on mathematics computation and mathematics vocabulary to perform word problems. It suggested that computation fluency will play an important role in mathematics achievement once the domain-specific information is memorized in older students. In addition, speed of domain-specific processing (retrieving basic facts and carrying) also predicts arithmetic performance in college students and non-college adults (Geary and Widaman 1987, 1992; Royer et al. 1999). 

Conversely, some studies were unable to establish a relationship between processing speed and mathematical achievement. Despite some research findings indicating processing speed to be an independent predictor of calculation (Bull and Johnston 1997), several others have reported that groups differing in calculation proficiency do not report any differences on measures of general processing speed (Andersson and Lyxell 2007; Jordan et al. 2003). Perceptual processing speed reported a substantial association with basic arithmetic ability, when assessed only by visual matching and pair cancellation tasks (Cowan et al. 2011; Fuchs et al. 2006; Lin 2020). However, no significant associations were found with algorithmic arithmetic (Fuchs et al. 2006) and word problem-solving (Lin 2020). A recent study found that information processing/decision speed, measured using the choice reaction time, is one of the key predictors of number sense, but it could not directly predict mathematical academic performance (Tikhomirova et al. 2020).

These inconsistent findings could be caused by the roles of different types of processing speed. Processing speed is divided into general processing speed (perceptual and decision speed) and specific processing speed (number facility/arithmetic and reading fluency [representing the academic fluency]) (Schneider and Mcgrew 2012). For example, the specific processing speed measured by number facility (Fuchs et al. 2006; Passolunghi and Lanfranchi 2012; Träff et al. 2017) or arithmetic fluency (Cowan et al. 2011; Geary 2011; Hecht et al. 2001; Lambert and Spinath 2017) can significantly predict mathematical achievement; however, this relationship is not shared by general processing speed (Andersson and Lyxell 2007; Fuchs et al. 2006; Jordan et al. 2003; Tikhomirova et al. 2020). 

To the best of our knowledge, no study has investigated whether general or specific processing speed differentially predicts mathematical achievement. In the present study, we employed a follow-up study to examine the effect of general or specific processing speed on mathematical achievement between the seventh and ninth grade. Given that specific processing speed involves symbolic representation and academic cognition, and general processing speed relies on basic cognitive processing. Previous studies also suggested the dissociated role of general and specific processing speed in arithmetic achievement (Geary and Widaman 1987; Widaman et al. 1992). Therefore, we hypothesized that specific or general processing speed would differentially predict the follow-up mathematical achievement. Specifically, we hypothesized that specific processing speed, rather than general processing speed, will predict mathematical achievements in junior high schools. Accordingly, we examined the role of specific processing speed in influencing mathematical achievement as well as other major academic achievements, because specific processing speed plays an important role in other academic performances (Berkowitz and Stern 2018; Zaboski et al. 2018).

## 2. Methods

### 2.1. Participants

All participating teenagers were recruited from a junior middle school in the Shijiazhuang municipality of Hebei Province in China. From Grades 7 to 9, 212 students (81 males and 131 females; mean age: 12.93 ± 0.58 years, ranging from 11.42 to 15.83) completed the cognitive tests in their first year, final examinations in Grade 7, and high school entrance examinations in Grade 9. In order to calculate the necessary sample size, we calculated it using G-power (Faul et al. 2009). “Linear multiple regression: Random model” in statistical test, and “A priori: Compute required sample size—given α, power, and effect size” in analysis type are chosen. We chose 0.086 as the effect size (H1), obtained after controlling age, gender, nonverbal matrix reasoning and word (Cui et al. 2017), α error probability is 0.05, power(1 − β error probability) is 0.8, number of predictors is 11. After calculation, we conclude that the minimum sample size is 192. We selected 212 students in this study, which is more than 192, so the number of participants is sufficient. 

**Figure 1:**
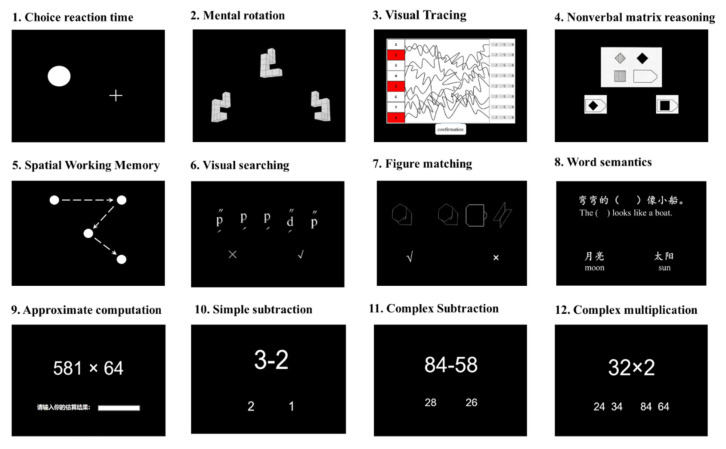
Display of a sample item for each test used in the current study.

All the participants were native Chinese speakers. The program, including the cognitive tests (See Figure 1), was fully explained to the students’ guardians (typically their parents). Subsequently, written informed consent was obtained from the guardians of all participants. All the utilised tests were fully explained to the counsellors; they also used students’ achievement scores and teachers’ subjective assessments to provide instructional suggestions for the students. The study was approved by the institutional review board (IRB) at the State Key Laboratory of Cognitive Neuroscience and Learning at Beijing Normal University. 

### 2.2. Cognitive Assessment

#### 2.2.1. Cognitive Tests

##### Nonverbal Matrix Reasoning

This task, a simplified version of Raven’s Progressive Matrices, aimed to evaluate students’ general intelligence (Raven and Court 1998). Each trial presented a large picture with a small ‘missing’ part in the upper part of the screen; two candidate pictures were located under the large picture. The participants had to choose one of the two candidate pictures that could be combined with the larger picture to form a whole picture. The test was limited to four minutes and contained 80 trials.

##### Mental Rotation

Mental rotation tasks are typically used for measuring spatial ability (Shepard and Metzler 1971). A three-dimensional figure is presented at the top of a screen in such tasks, with two candidate figures at the bottom of the screen. The rotation angles for the matching figure ranged from 15°–345°. Participants were asked to choose the figure that would be rotated from the top by pressing ‘Q’ or ‘P’ on the keyboard. This test contained 180 trials with 3-min time limits for each. 

##### Spatial Working Memory

This task, which measured spatial working memory ability, was adapted from the Corsi Blocks Task (Corsi 1972). White dots were displayed on the screen in an implicit 3 × 3 lattice, and the time interval between the points consisted of 1000 ms. Students had to click on the screen using the mouse in the order of their appearance, and the number of points varied from 4 to 9. We chose the distance as an indicator (that is, the distance between the position of the point’s appearance and the position where the students clicked). After all the dots were presented, the participants used a mouse to click on the lattice based on the position and order of the dot presentation. A smaller distance indicated a higher score. 

##### Visual Tracing

The visual tracing task was adapted from Groffman’s visual tracing test and was designed to examine visual attention ability (Groffman 1994). The centre of the computer screen had 6–9 curves that were interwoven in a left to right direction. Participants had to track the lines that started from the target number with red or black squares on the left with their eyes; furthermore, they had to select the corresponding number at the end of the line on the right. Notably, they were allowed to use only their eyes to track the lines—not fingers or other tools. This task contained 12 trials. 

##### Visual Searching

Visual searching tasks employ a d2 test paradigm, and they are commonly used for evaluating visual attention (Bates and Lemay 2004). The letters ‘d’ and ‘p’ were presented on the screen surrounded by one to four dashes each. Students were required to judge whether there was a letter ‘d’ with 2 dashes; furthermore, they had to ignore the position of the dashes (both above the ‘d’, both below the ‘d’, or one above and one below the ‘d’). Participants were asked to press the ‘Q’ key when one target appeared in the stimuli, or the ‘P’ key when there was no target.

#### 2.2.2. General Processing Speed

##### Choice Reaction Time

This task evaluated students’ mental processing speed (Butterworth 2003). Participants were asked to press certain keys on the keyboard every time they saw a white dot on the screen; furthermore, they were asked to press the ‘Q’ key every time the white dot appeared to the left of the fixation cross and press the ‘P’ key when the dot was located to the right of the fixation cross. This test included 30 randomly presented trials on the screen (half of the trials that included the dot on the left). The response-stimulus interval varied randomly between 1500 and 3000 ms. 

##### Figure Matching

The figure matching test was used for measuring perception speed ability (Cheng et al. 2020; Zhou and Cheng 2015; Zhou et al. 2015). It was adapted from the identical picture test in the Manual for the Kit of Factor-Referenced Cognitive Tests (Ekstrom et al. 1976). In each trial, four pictures were presented in the centre of the screen: one target picture on the left side and three candidate pictures on the right. Participants had to judge whether the picture on the left was identical to any of the pictures on the right (60 matched trials and 60 non-matched trials); if it was, they were required to press the ‘Q’ key; however, if it was not, they had to press the ‘P’ key. This task contained 120 trials. Participants had to complete all trials within three 40-trial sessions.

#### 2.2.3. Specific Processing Speed

##### Word Semantics (Reading Fluency)

This task was used for evaluating students’ semantic comprehension and semantic memory with regard to language ability (Mummery et al. 1998). The task materials were adapted from textbooks used in primary, middle, and high schools in China. Each trial featured a sentence on the screen, which had a missing word with two alternative words at the bottom. Participants had to choose a word that could accord with the sentence meaning by pressing either the ‘Q’ key or the ‘P’ key. The stimulus stayed on the screen until the participant had responded. The test was limited to five minutes.

##### Simple Subtraction (Arithmetic Fluency)

This task contains 92 trials, which are simple subtraction problems. The largest minuend was 18, and the smallest one was 2. The difference between the two operands was always a single digit, and the minuend is integers up to 18. Two choices were provided at the bottom, with one of them being the correct answer; the other was an incorrect answer, which deviated from 3 (i.e., ±1, ±2, or ±3). The time limit was two minutes.

##### Complex Subtraction (Arithmetic Fluency)

The complex subtraction task contains 96 problems involving two-digit operands. Two-digit subtraction formulae were presented in the centre of a screen having two candidate answers (i.e., incorrect and correct answers) beneath each problem. Each incorrect answer was within ±10 values of the correct answer. Participants were instructed to select the correct answer by pressing either the ‘Q’ key or the ‘P’ key. The task time limit was two minutes.

##### Complex Multiplication (Arithmetic Fluency)

The multiple-digit multiplication task contained 76 problems involving multiplication of a two-digit number by a one-digit number (e.g., 34 × 3). Each trial featured a multiple-digit multiplication problem that was presented in the centre of a screen with four candidate answers (i.e., one correct answer and three false answers) beneath the problem. The four candidate answers were divided into two groups with each group having two answers. Participants had to determine the group that had the correct answer by pressing the ‘Q’ or ‘P’ key. The task limit was two minutes.

### 2.3. Academic Achievement

For the 7th grade, other than the aforementioned cognitive test results, the school provided academic achievement scores for major subjects (i.e., Mathematics, Chinese, English) from the final examination of the semester. The achievement test was developed by the Instruction Research Unit (affiliated to the local Department of Education) and administered to all students in the district at the end of each semester. 

For the 9th grade, the school provided the academic achievement scores for major subjects (i.e., Mathematics, Chinese, English) from the senior high school entrance examination. This achievement test was also developed by the Instruction Research Unit (affiliated to the local Department of Education).

### 2.4. Procedure

The task battery was administered using two 45 min sessions. Testing was applied to groups of participants (one class at a time) in computer classrooms. Six to seven experimenters and one class teacher monitored each class. All the students experienced the tasks following the same order. During each task, instructions were provided, and a practice session was completed prior to the formal testing. Each task’s practice consisted of four to six trials that were similar to those used in the formal testing. During the practice trials, the computer screen provided students with feedback; the feedback for correct responses was ‘Correct! Can you go faster?’, and the feedback for incorrect responses was ‘It is wrong. Try again.’ Students were allowed to question the experimenters during the practice sessions. After all students in a given class had completed the practice session and indicated that they had no more questions for the experimenters, the main experimenter would say, ‘Start’, and the students would press any key to begin the formal testing.

For all but two tasks, the students responded by pressing keys on a computer keyboard. For the visual tracing and spatial working memory task, the students used a mouse to mark the correct answer. Overall, 14 cognitive tests were administered using a Web-based psychological system on 30 June 2014 (i.e., www.dweipsy.com/lattice) (Cheng et al. 2013, 2020; Wei et al. 2012). All data were collected in March 2014. 

### 2.5. Data Analysis

Except for two tasks (i.e., choice reaction time and spatial working memory), performance on all the cognitive tests were evaluated using corrected scores calculated by subtracting the number of incorrect responses from the number of correct responses in order to control for guessing effect (Cirino 2011). For the choice reaction time test, we calculated the median reaction time. For spatial working memory, we calculated the response accuracy. Descriptive statistics were performed for all the tests. We performed Pearson’s correlation analyses to investigate the relationships between all measures in the cognitive tests and academic performances. Moreover, we conducted hierarchical regression analyses to examine the role of general processing speed, including perceptual speed (figure matching) and decision speed (choice reaction time); and specific processing speed, including reading fluency (word semantic) and arithmetic fluency (simple subtraction, complex subtraction, and complex multiplication) in academic achievements while controlling for age, gender, and all other types of general cognitive abilities. 

## 3. Results

### 3.1. Correlation Analysis

Table 1 shows the descriptive statistics and correlation coefficients of academic achievements and cognitive abilities. All cognitive tests had acceptable reliabilities, ranging from 0.78 to 0.93. A Bonferroni correction was used for maintaining the *p*-value < 0.05 across the 136 correlations in Table 1. Thus, a conservative *p*-value of < 0.00037 (=0.05/136) was considered statistically significant. The intercorrelations between all academic measures were significant (all were *p* < 0.0003). All the specific processing speed measures (word semantic, simple subtraction, complex subtraction, and complex multiplication) were significantly correlated to all academic measures.

### 3.2. Regression Analysis

To examine the associations of general and specific processing speed with mathematical achievements, we performed multiple hierarchical regression analyses (see Table 2). All the regression models used Bonferroni corrections, and an adjusted alpha value of 0.05 corresponded to 0.005 before the correction (0.05/10 = 0.005). Hierarchical regression analyses showed that, after controlling for age, gender, nonverbal matrix reasoning, spatial abilities (mental rotation, spatial working memory) and visual attention (visual tracing, visual searching), specific processing speed including reading fluency (word semantic) and arithmetic fluency (simple subtraction and complex subtraction) made a significant unique contribution to mathematical achievement in the 7th grade. Word semantic, simple subtraction, and complex subtraction accounted for 5.6%, 7.2%, and 5.1% of the variances in mathematics. Specific processing speed also significantly contributed to Chinese and English achievements in the 7th grade, where it accounted for at least 4.2% of the variance in academic achievements. More detailed results of regression analysis were also showed in Supplementary Materials.

In the 9th grade, specific processing speed including word semantic, simple subtraction, and complex subtraction could predict mathematical achievements, and they accounted for 4.4%, 5.4% and 5.4% of the variances in mathematical achievement in the high school entrance examination. Specific processing speed including word semantic, simple subtraction, and complex subtraction could also significantly predict Chinese and English academic achievements, thus accounting for at least 4.5% of the variance in high school entrance examination achievements. However, complex multiplication could not account for the variances in mathematical achievement, even after controlling for age, gender, nonverbal matrix reasoning, spatial abilities, and visual attention.

After controlling for age, gender, nonverbal matrix reasoning, spatial abilities (mental rotation, spatial working memory), and visual attention (visual tracing, visual searching), complex multiplication predicted variances in Chinese (Δ*R*^2^ = 0.033, *p* < 0.05) and English (Δ*R*^2^ = 0.034, *p* < 0.05) in Grade 7 and Chinese (Δ*R*^2^ = 0.029, *p* < 0.05), English (Δ*R*^2^ = 0.039, *p* < 0.01), and total scores (Δ*R*^2^ = 0.033, *p* < 0.05) in Grade 9. Therefore, the prediction power of academic processing speed for mathematical achievements decreased with the reduction in arithmetic fluency (from simple and complex subtractions to complex multiplication). 

## 4. Discussion

This study examined the role of general and specific processing speed as predictors of mathematical achievements. Furthermore, it investigated the generalisation of these predictions among other academic achievements of major subjects. The results showed that specific processing speed—reading fluency (word semantic) and arithmetic fluency (simple subtraction and complex subtraction)—rather than general processing speed made a significant unique contribution to mathematical achievements in Grade 7, and it predicted mathematical achievements in the 9th grade high school entrance examinations. Meanwhile, academic processing speed could also significantly contribute to the academic achievements of Chinese and English in Grade 7 and Grade 9. These results suggest that specific processing speed, rather than general processing speed, could predict mathematical achievement; furthermore, this prediction could be generalised to academic achievements of major subjects among junior high school students. 

Thus, in line with previous research, the present study illustrated the importance of specific processing speed in mathematical achievement for junior school students (Cowan et al. 2011; Geary 2011; Hecht et al. 2001; Lambert and Spinath 2017). First, the predictive role of reading fluency for mathematical achievement might reflect the influence of reading-related processes (e.g., semantic comprehension) on mathematical word problem solving. Mathematical concept comprehension—requiring the application of conceptual and semantic knowledge—and word problem solving are essential competencies among junior high school students, whose mathematics curriculum mainly includes algebra and geometry (Geary et al. 2015; Zhang et al. 2012). In addition, some studies also found that reading comprehension (Cowan et al. 2005), processing speed (Swanson and Beebe-Frankenberger 2004) and reading fluency (Fuchs et al. 2021) is related to math problem-solving. The prediction of word semantic to academic achievement showed the important role of specific processing speed or reading fluency in future academic achievement. 

Second, the predictive role of arithmetic fluency for mathematics achievement revealed the essential role of simple arithmetic in mathematics curriculum of junior high school. Simple arithmetic involves a higher amount of arithmetic facts retrieval than complex multiplication, which often employs strategy-based calculation (Li et al. 2019). For example, the answers to two-digit multiplication problems such as 11 × 9 can be calculated using a procedural strategy (i.e., 11 × 9 = 99). In contrast, simple arithmetic is characterized by fluency processing of specific symbolic representation (Cheng et al. 2017; Zhou et al. 2007; Zhou and Dong 2003). For example, students use fact retrieval strategies for simple subtraction operations (e.g., 16 − 7 = 9). Compared to complex multiplication, simple arithmetic more rely on arithmetic facts retrieval involved symbolic fluency processing. A recent study also demonstrated that computation fluency is an essential predictor of word problem solving across younger and older students (Lin 2020). Fluent academic knowledge (i.e., simple arithmetic facts retrieval) could help in efficiently allocating cognitive resources to solve more complex problems. 

In line with previous research (McGrew and Wendling 2010), the present study illustrated the importance of processing speed in determining academic achievement. Furthermore, specific processing speed involves a speed-based operation for specific symbolic, arithmetic facts or semantic memory; therefore, it differs from general processing speed, which involves visual perception and decision speed. Specific processing speed reflected the academic fluency of specific symbolic representation. In this study, we found that specific processing speed could still predict academic achievement for a period of three years after controlling for general cognitive abilities (nonverbal matrix reasoning, spatial abilities, visual attention). Similarly, the fluency of processing basic numerical relationships bridge the codevelopment of reading and mathematics achievement (Spencer et al. 2021). It is possible that the correlation between specific processing speed and academic achievement emerge because the same underlying brain systems (e.g., hippocampal-neocortical functional connectivity) support rapid retrieval of domain-specific information and academic learning (Qin et al. 2014; Schlaggar and McCandliss 2007). This finding suggested that academic fluency had a stable effect on mathematical achievement. 

Moreover, the current study found that in addition to mathematics, exact arithmetic could also predict other academic achievements of major subjects. In junior middle school, students accumulate subject knowledge through all kinds of symbols based on mathematics or language. Consensus holds that mathematics and language abilities form the cornerstone of academic achievements (Zaboski et al. 2018). Symbol-related academic fluency could help junior students quickly resolve problems related to subject learning. Thus, symbolic arithmetic and reading fluency is frequently used to resolve problems in various subjects. Academic fluency was not specific to mathematics, rather, it was considered to be generalised to all academic achievements. The follow-up findings suggested that specific processing speed had a strong effect on academic achievement. 

However, the present study has some limitations. First, we did not analyze the effect of gender differences on the role of processing speed in academic achievements. Hierarchical regression analyses showed that in the first step, age and gender differences could significantly predict academic achievements expected for the 7th grade mathematics. Gender was served as the control variable. Future study could focus on gender differences in the effect of general processing speed on academic achievements. Second, a word semantic test was used to assess reading fluency, and simple subtraction, complex subtraction and complex multiplication was used to assess arithmetic fluency. These tasks are typical assessment tests, and all participants were capable of performing these tasks. However, it would be better if future research employed more tests per domain to assess specific processing speed. 

## 5. Conclusions

In summary, this study extends previous findings by revealing the differential role of general and specific processing speed in predicting mathematical achievements. The results demonstrated that specific processing speed, rather than general processing speed, predicted future mathematical achievements, and that this prediction could be generalised to other academic achievements of major subjects. Thus, we concluded that specific processing speed reflected academic fluency, which had a strong prediction power for high school entrance examination achievements. Therefore, in the junior school education context, teachers of all subjects should pay attention to teaching methods and contents related to academic fluency. Future studies should clarify the effect of academic fluency training on junior high school-aged students’ academic performances. 

## Figures and Tables

**Table 1:** Descriptive statistics and correlations of cognitive abilities and academic achievement among all participants.

Tests	Mean (SD)	Split-Half Reliability	1	2	3	4	5	6	7	8	9	10	11	12	13	14	15	16
1. Nonverbal matrix reasoning	20.46 (7.50)	0.78	-															
2. Mental rotation	20.15 (9.76)	0.86	0.24 **	-														
3. Spatial working memory	82.24 (7.33)	0.91	0.07	0.23 **	-													
4. Visual tracing	19.32 (5.12)	0.92	0.25 **	0.29 **	0.13	--												
5. Visual search	30.65 (25.09)	0.92	0.25 **	0.17 *	0.09	0.16 *	--											
6. Figure matching	53.06 (24.98)	0.92	0.21 **	0.34 **	0.20 **	0.26 **	0.32 **	--										
7. Choice reaction time	319.94 (59.16)	0.93	−0.11	−0.01	−0.11	−0.19 **	0.03	−0.14 *	--									
8. Word semantic	32.93 (7.44)	0.82	0.23 **	0.12	0.07	0.26 **	0.20 **	0.14 *	−0.19 **	--								
9. Simple subtraction	44.04 (11.15)	0.86	0.23 **	0.12	0.12	0.15 *	0.39 **	0.42 **	−0.04	0.24 **	--							
10. Complex subtraction	21.40 (9.12)	0.85	0.18 **	0.08	0.08	0.13	0.43 **	0.34 **	−0.07	0.13	0.47 **	--						
11. Complex multiplication	29.02 (6.78)	0.78	0.14 *	0.08	0.43 **	0.18 **	0.26 **	0.27 **	−0.22 **	0.11	0.39 **	0.21 **	--					
12. Mathematics in Grade 7	81.40 (18.03)	N/A	0.35 **	0.24 **	0.10	0.31 **	0.39 **	0.29 **	−0.14	0.42 **	0.46 **	0.41 **	0.28 **	--				
13. Chinese in Grade 7	83.72 (8.27)	N/A	0.25 **	0.11	0.13	0.28 **	0.26 **	0.20 **	−0.25 **	0.45 **	0.45 **	0.37 **	0.31 **	0.66 **	--			
14. English in Grade 7	84.86 (15.35)	N/A	0.33 **	0.12	0.09	0.25 **	0.41 **	0.20 **	−0.18 **	0.41 **	0.51 **	0.46 **	0.33 **	0.67 **	0.78 **	--		
15. Chinese in Grade 9	101.32 (10.66)	N/A	0.36 **	0.16 *	0.13	0.38 **	0.30 **	0.18 **	−0.31 **	0.49 **	0.51 **	0.38 **	0.34 **	0.77 **	0.75 **	0.74 **	--	
16. Mathematics in Grade 9	89.25 (21.60)	N/A	0.34 **	0.21 **	0.10	0.29 **	0.37 **	0.24 **	−0.16 *	0.39 **	0.43 **	0.41 **	0.26 **	0.67 **	0.87 **	0.75 **	0.77 **	--
17. English in Grade 9	89.94 (24.62)	N/A	0.23 **	0.10	0.09	0.24 **	0.38 **	0.21 **	−0.18 **	0.43 **	0.47 **	0.44 **	0.31 **	0.66 **	0.77 **	0.86 **	0.76 **	0.83 **

* *p* < 0.05, ** *p* < 0.01, corrected with Bonferroni correction method for all correlation analyses. SD: Standard Deviation; N/A: Not applicable.

**Table 2:** Hierarchical regression models predicting academic achievement in Grades 7 and 9 of junior high school considering age, gender, cognitive tests, and general and specific processing speed.

Predictors	Grade 7			Grade 9		
	Mathematics	Chinese	English	Mathematics	Chinese	English
	∆R^2^	∆R^2^	∆R^2^	∆R^2^	∆R^2^	∆R^2^
Step 1 Age, Gender	0.043	0.081 **	0.138 **	0.060 *	0.080 **	0.137 **
Step 2 Nonverbal matrix reasoning	0.114 **	0.053 **	0.094 **	0.105 **	0.118 **	0.043 *
Step 3 Spatial abilities (mental rotation, Spatial working memory)	0.036	0.015	0.009	0.027	0.015	0.011
Step 4 Visual attention (visual tracing, visual search)	0.098 **	0.068 **	0.099 **	0.084 **	0.102 **	0.096 **
*General processing speed*						
Step 5 Choice reaction time	0.009	0.008	0.003	0.003	0.000	0.007
Step 5 Figure matching	0.006	0.038 *	0.021	0.013	0.058 **	0.022
*Specific processing speed*						
Step 5 Word semantic	0.056 **	0.081 **	0.042 **	0.044 **	0.082 **	0.063 **
Step 5 Simple subtraction	0.072 **	0.091 **	0.088 **	0.054 **	0.109 **	0.080 **
Step 5 Complex subtraction	0.051 **	0.057 **	0.066 **	0.054 **	0.045 **	0.068 **
Step 5 Complex multiplication	0.020	0.033 *	0.034 *	0.014	0.039 **	0.033 *

Note: The alpha values are set to 0.05/10 = 0.005, 0.01/10 = 0.001. * *p* < 0.05, with Bonferroni correction. ** *p* < 0.01, with Bonferroni correction.

## Data Availability

The data are currently not publicly available due to participant privacy, but they are available from the corresponding author upon reasonable request. Every request will be reviewed by the institutional review board of the State Key Laboratory of Cognitive Neuroscience and Learning at Beijing Normal University.

## Supplementary Materials

The following are available online at https://www.mdpi.com/article/10.3390/jintelligence10010001/s1, Table S1. Hierarchical regression models predicting academic achievement in Grades 7 of junior high school considering age, gender, cognitive tests, general processing speed (Choice reaction time), Table S2. Hierarchical regression models predicting academic achievement in Grades 7 of junior high school considering age, gender, cognitive tests, general processing speed (Figure matching), Table S3. Hierarchical regression models predicting academic achievement in Grades 7 of junior high school considering age, gender, cognitive tests, specific processing speed (Word semantics), Table S4. Hierarchical regression models predicting academic achievement in Grades 7 of junior high school considering age, gender, cognitive tests, specific processing speed (Simple subtraction), Table S5. Hierarchical regression models predicting academic achievement in Grades 7 of junior high school considering age, gender, cognitive tests, specific processing speed (Complex subtraction), Table S6. Hierarchical regression models predicting academic achievement in Grades 7 of junior high school considering age, gender, cognitive tests, specific processing speed (Complex multiplication), Table S7. Hierarchical regression models predicting academic achievement in Grades 9 of junior high school considering age, gender, cognitive tests, general processing speed (Choice reaction time), Table S8. Hierarchical regression models predicting academic achievement in Grades 9 of junior high school considering age, gender, cognitive tests, general processing speed (Figure matching), Table S9. Hierarchical regression models predicting academic achievement in Grades 9 of junior high school considering age, gender, cognitive tests, specific processing speed (Word semantics), Table S10. Hierarchical regression models predicting academic achievement in Grades 9 of junior high school considering age, gender, cognitive tests, specific processing speed (Simple subtraction). Table S11. Hierarchical regression models predicting academic achievement in Grades 9 of junior high school considering age, gender, cognitive tests, specific processing speed (Complex subtraction). Table S12. Hierarchical regression models predicting academic achievement in Grades 9 of junior high school considering age, gender, cognitive tests, specific processing speed (Complex multiplication).
