# Supplementary material for: Examining the Differential Role of General and Specific Processing Speed in Predicting Mathematical Achievement in Junior High School
Source: J Intell. 2021 Dec 21;10(1):1. doi: 10.3390/jintelligence10010001 (PMC8788420; doi:10.3390/jintelligence10010001)
Supplement: Supplementary file 1 [file jintelligence-10-00001-s001.zip › jintelligence-1437635-supplementary-update.pdf]

Journal of
Intelligence

Article

Examining the Differential Role of General and Specific Processing Speed in Predicting Mathematical Achievement in Junior High School

Dazhi Cheng [1,2,3,4], Kaihui Shi [1,3], Naiyi Wang [2], Xinyang Miao [1,3,4] and Xinlin Zhou [1,3,*]

[1] State Key Laboratory of Cognitive Neuroscience and Learning, IDG/McGovern Institute for Brain Research, Beijing Normal University, Beijing 100875, China; chengdazhi1015@126.com (D.C.); shikaihui0103@foxmail.com (K.S.); xmiao97@hotmail.com (X.M.)
[2] Lab for Educational Neuroscience, Center for Educational Science and Technology, Faculty of Education, Beijing Normal University, Beijing 100875, China; wangnaiyi@bnu.edu.cn
[3] Advanced Innovation Center for Future Education, Beijing Normal University, Beijing 100875, China
[4] Department of Pediatric Neurology, Capital Institute of Pediatrics, Beijing 100020, China
* Correspondence: zhou_xinlin@bnu.edu.cn

Table S1. Hierarchical regression models predicting academic achievement in Grades 7 of junior high school considering age, gender, cognitive tests, general processing speed (Choice reaction time).

Predictors.	Mathematics .					Chinese.					English.				
	Step 1	Step 2	Step 3	Step 4	Step 5	Step 1	Step 2	Step 3	Step 4	Step 5	Step 1	Step 2	Step 3	Step 4	Step 5
	β	*β*	*β*	*β*	*β*	*β*	*β*	*β*	*β*	*β*	*β*	*β*	*β*	*β*	*β*
Age(months)	-0.074	-0.049	-0.044	-0.026	-0.023	0.013	0.030	0.030	0.059	0.066	-0.006	0.017	0.018	0.033	0.039
Gender	0.192*	0.173	0.198*	0.158	0.160	0.285**	0.272**	0.279**	0.262**	0.268**	0.371**	0.354**	0.365**	0.322**	0.326**
Nonverbal matrix reasoning		0.340**	0.291**	0.204*	0.195*		0.232**	0.206*	0.136	0.117		0.308**	0.285**	0.197*	0.183*
Mental rotation			0.19*	0.116	0.124			0.084	0.017	0.035			0.088	0.015	0.028
Spatial working memory			0.023	0.000	-0.010			0.075	0.051	0.029			0.028	0.005	-0.011
Visual tracing				0.184*	0.167				0.230*	0.191				0.169	0.140
Visual search				0.262**	0.269**				0.138	0.153				0.274**	0.29**
Choice reaction time					-0.092					-0.209*					-0.154
	ΔR^2=0.043	ΔR^2=0.114**	ΔR^2=0.036	ΔR^2=0.098**	ΔR^2=0.008	ΔR^2=0.081**	ΔR^2=0.053**	ΔR^2=0.015	ΔR^2=0.068**	ΔR^2=0.041*	ΔR^2=0.138**	ΔR^2=0.094**	ΔR^2=0.009	ΔR^2=0.099**	ΔR^2=0.022

Note: The alpha values are set to 0.05/10 = 0.005, 0.01/10 = 0.001. **p* < 0.05, with Bonferroni correction. ***p* < 0.01, with Bonferroni correction.

Table S2. Hierarchical regression models predicting academic achievement in Grades 7 of junior high school considering age, gender, cognitive tests, general processing speed (Figure matching).

Predictors	Mathematics					Chinese					English				
	Step 1	Step 2	Step 3	Step 4	Step 5	Step 1	Step 2	Step 3	Step 4	Step 5	Step 1	Step 2	Step 3	Step 4	Step 5
	β	β	β	β	β	β	β	β	β	β	β	β	β	β	β
Age(months)	-0.074	-0.049	-0.044	-0.026	-0.018	0.013	0.030	0.030	0.059	0.066	-0.006	0.017	0.018	0.033	0.038
Gender	0.192*	0.173	0.198*	0.158	0.167	0.285**	0.272**	0.279**	0.262**	0.271**	0.371**	0.354**	0.365**	0.322**	0.327**
Nonverbal matrix reasoning		0.340**	0.291**	0.204*	0.197*		0.232**	0.206*	0.136	0.129		0.308**	0.285**	0.197*	0.193*
Mental rotation			0.19*	0.116	0.093			0.084	0.017	-0.004			0.088	0.015	0.002
Spatial working memory			0.023	0.000	-0.014			0.075	0.051	0.039			0.028	0.005	-0.002
Visual tracing				0.184*	0.172				0.230*	0.219*				0.169	0.162
Visual search				0.262**	0.234**				0.138	0.113				0.274**	0.259**
Figure matching					0.110					0.099					0.060
	ΔR^2=0.043	ΔR^2=0.114**	ΔR^2=0.036	ΔR^2=0.098**	ΔR^2=0.009	ΔR^2=0.081**	ΔR^2=0.053**	ΔR^2=0.015	ΔR^2=0.068**	ΔR^2=0.008	ΔR^2=0.138**	ΔR^2=0.094**	ΔR^2=0.009	ΔR^2=0.099**	ΔR^2=0.003

Note: The alpha values are set to 0.05/10 = 0.005, 0.01/10 = 0.001. *p < 0.05, with Bonferroni correction. **p < 0.01, with Bonferroni correction.

Table S3. Hierarchical regression models predicting academic achievement in Grades 7 of junior high school considering age, gender, cognitive tests, specific processing speed (Word semantics).

Predictors	Mathematics					Chinese					English				
	Step 1	Step 2	Step 3	Step 4	Step 5	Step 1	Step 2	Step 3	Step 4	Step 5	Step 1	Step 2	Step 3	Step 4	Step 5
	β	*β*	*β*	*β*	*β*	*β*	*β*	*β*	*β*	*β*	*β*	*β*	*β*	*β*	*β*
Age(months)	-0.074	-0.049	-0.044	-0.026	-0.024	0.013	0.030	0.030	0.059	0.061	-0.006	0.017	0.018	0.033	0.035
Gender	0.192*	0.173	0.198*	0.158	0.097	0.285**	0.272**	0.279**	0.262**	0.189*	0.371**	0.354**	0.365**	0.322**	0.269**
Nonverbal matrix reasoning		0.340**	0.291**	0.204*	0.168		0.232**	0.206*	0.136	0.093		0.308**	0.285**	0.197*	0.166
Mental rotation			0.19*	0.116	0.105			0.084	0.017	0.004			0.088	0.015	0.006
Spatial working memory			0.023	0.000	-0.001			0.075	0.051	0.051			0.028	0.005	0.005
Visual tracing				0.184*	0.130				0.230*	0.165				0.169	0.121
Visual search				0.262**	0.242**				0.138	0.113				0.274**	0.256**
Word semantics					0.258**					0.312**					0.226**
	ΔR^2=0.043	ΔR^2=0.114**	ΔR^2=0.036	ΔR^2=0.098**	ΔR^2=0.056**	ΔR^2=0.081**	ΔR^2=0.053**	ΔR^2=0.015	ΔR^2=0.068**	ΔR^2=0.081**	ΔR^2=0.138**	ΔR^2=0.094**	ΔR^2=0.009	ΔR^2=0.099**	ΔR^2=0.043**

Note: The alpha values are set to 0.05/10 = 0.005, 0.01/10 = 0.001. *$p < 0.05$, with Bonferroni correction. **$p < 0.01$, with Bonferroni correction.

Table S4. Hierarchical regression models predicting academic achievement in Grades 7 of junior high school considering age, gender, cognitive tests, specific processing speed (Simple subtraction).

Predictors	Mathematics					Chinese					English				
	Step 1	Step 2	Step 3	Step 4	Step 5	Step 1	Step 2	Step 3	Step 4	Step 5	Step 1	Step 2	Step 3	Step 4	Step 5
	β	β	β	β	β	β	β	β	β	β	β	β	β	β	β
Age(months)	-0.074	-0.049	-0.044	-0.026	-0.026	0.013	0.030	0.030	0.059	0.059	-0.006	0.017	0.018	0.033	0.034
Gender	0.192*	0.173	0.198*	0.158	0.117	0.285**	0.272**	0.279**	0.262**	0.217**	0.371**	0.354**	0.365**	0.322**	0.277**
Nonverbal matrix reasoning		0.340**	0.291**	0.204*	0.167*		0.232**	0.206*	0.136	0.094		0.308**	0.285**	0.197*	0.157
Mental rotation			0.19*	0.116	0.108			0.084	0.017	0.008			0.088	0.015	0.007
Spatial working memory			0.023	0.000	-0.017			0.075	0.051	0.033			0.028	0.005	-0.013
Visual tracing				0.184*	0.165				0.230*	0.209				0.169	0.148
Visual search				0.262**	0.167				0.138	0.031*				0.274**	0.169*
Simple subtraction					0.300**					0.338**					0.332**
	ΔR^2=0.043	ΔR^2=0.114**	ΔR^2=0.036	ΔR^2=0.098**	ΔR^2=0.072**	ΔR^2=0.081**	ΔR^2=0.053**	ΔR^2=0.015	ΔR^2=0.068**	ΔR^2=0.091**	ΔR^2=0.138**	ΔR^2=0.094**	ΔR^2=0.009	ΔR^2=0.099**	ΔR^2=0.088**

Note: The alpha values are set to 0.05/10 = 0.005, 0.01/10 = 0.001. *$p < 0.05$, with Bonferroni correction. **$p < 0.01$, with Bonferroni correction.

Table S5. Hierarchical regression models predicting academic achievement in Grades 7 of junior high school considering age, gender, cognitive tests, specific processing speed (Complex subtraction).

Predictors	Mathematics					Chinese					English				
	Step 1	Step 2	Step 3	Step 4	Step 5	Step 1	Step 2	Step 3	Step 4	Step 5	Step 1	Step 2	Step 3	Step 4	Step 5
	β	*β*	*β*	*β*	*β*	*β*	*β*	*β*	*β*	*β*	*β*	*β*	*β*	*β*	*β*
Age(months)	-0.07	-0.05	-0.04	-0.03	-0.02	0.01	0.03	0.03	0.06	0.07	-0.01	0.02	0.02	0.03	0.04
Gender	0.192*	0.17	0.198*	0.16	0.13	0.285**	0.272**	0.279**	0.262**	0.237**	0.371**	0.354**	0.365**	0.322**	0.295**
Nonverbal matrix reasoning		0.340**	0.291**	0.204*	0.188*		0.232**	0.206*	0.14	0.12		0.308**	0.285**	0.197*	0.179*
Mental rotation			0.19*	0.12	0.12			0.08	0.02	0.02			0.09	0.01	0.02
Spatial working memory			0.02	0.00	-0.01			0.07	0.05	0.04			0.03	0.01	0.00
Visual tracing				0.184*	0.17				0.230*	0.22				0.17	0.15
Visual search				0.262**	0.16				0.14	0.03				0.274**	0.16
Complex subtraction					0.254**					0.267**					0.292**
	ΔR^2=0.043	ΔR^2=0.114**	ΔR^2=0.036	ΔR^2=0.098**	ΔR^2=0.051**	ΔR^2=0.081**	ΔR^2=0.053**	ΔR^2=0.015	ΔR^2=0.068**	ΔR^2=0.057**	ΔR^2=0.138**	ΔR^2=0.094**	ΔR^2=0.009	ΔR^2=0.099**	ΔR^2=0.068**

Note: The alpha values are set to 0.05/10 = 0.005, 0.01/10 = 0.001. **p* < 0.05, with Bonferroni correction. ***p* < 0.01, with Bonferroni correction.

Table S6. Hierarchical regression models predicting academic achievement in Grades 7 of junior high school considering age, gender, cognitive tests, specific processing speed (Complex multiplication).

Predictors	Mathematics					Chinese					English				
	Step 1	Step 2	Step 3	Step 4	Step 5	Step 1	Step 2	Step 3	Step 4	Step 5	Step 1	Step 2	Step 3	Step 4	Step 5
	β	*β*	*β*	*β*	*β*	*β*	*β*	*β*	*β*	*β*	*β*	*β*	*β*	*β*	*β*
Age(months)	-0.07	-0.05	-0.04	-0.03	-0.03	0.01	0.03	0.03	0.06	0.05	-0.01	0.02	0.02	0.03	0.02
Gender	0.192*	0.17	0.198*	0.16	0.15	0.285**	0.272**	0.279**	0.262**	0.253**	0.371**	0.354**	0.365**	0.322**	0.313**
Nonverbal matrix reasoning		0.340**	0.291**	0.204*	0.193*		0.232**	0.206*	0.14	0.12		0.308**	0.285**	0.197*	0.18
Mental rotation			0.19*	0.12	0.13			0.08	0.02	0.03			0.09	0.01	0.03
Spatial working memory			0.02	0.00	-0.07			0.07	0.05	-0.04			0.03	0.01	-0.08
Visual tracing				0.184*	0.17				0.230*	0.206*				0.17	0.14
Visual search				0.262**	0.231**				0.14	0.10				0.274**	0.234**
Complex multiplication					0.16					0.214*					0.211*
	ΔR^2=0.043	ΔR^2=0.114**	ΔR^2=0.036	ΔR^2=0.098**	ΔR^2=0.020	ΔR^2=0.081**	ΔR^2=0.053**	ΔR^2=0.015	ΔR^2=0.068**	ΔR^2=0.034*	ΔR^2=0.138**	ΔR^2=0.094**	ΔR^2=0.009	ΔR^2=0.099**	ΔR^2=0.033*

Note: The alpha values are set to 0.05/10 = 0.005, 0.01/10 = 0.001. **p* < 0.05, with Bonferroni correction. ***p* < 0.01, with Bonferroni correction.

Table S7. Hierarchical regression models predicting academic achievement in Grades 9 of junior high school considering age, gender, cognitive tests, general processing speed (Choice reaction time).

Predictors	Mathematics					Chinese					English				
	Step 1	Step 2	Step 3	Step 4	Step 5	Step 1	Step 2	Step 3	Step 4	Step 5	Step 1	Step 2	Step 3	Step 4	Step 5
	β	*β*	*β*	*β*	*β*	*β*	*β*	*β*	*β*	*β*	*β*	*β*	*β*	*β*	*β*
Age(months)	-0.074	-0.049	-0.044	-0.026	-0.023	0.013	0.030	0.030	0.059	0.066	-0.006	0.017	0.018	0.033	0.039
Gender	0.192*	0.173	0.198*	0.158	0.160	0.285**	0.272**	0.279**	0.262**	0.268**	0.371**	0.354**	0.365**	0.322**	0.326**
Nonverbal matrix reasoning		0.340**	0.291**	0.204*	0.195*		0.232**	0.206*	0.136	0.117		0.308**	0.285**	0.197*	0.183*
Mental rotation			0.19*	0.116	0.124			0.084	0.017	0.035			0.088	0.015	0.028
Spatial working memory			0.023	0.000	-0.010			0.075	0.051	0.029			0.028	0.005	-0.011
Visual tracing				0.184*	0.167				0.230*	0.191				0.169	0.140
Visual search				0.262**	0.269**				0.138	0.153				0.274**	0.29**
Choice reaction time					-0.092					-0.209*					-0.154
	ΔR^2=0.043	ΔR^2=0.114**	ΔR^2=0.036	ΔR^2=0.098**	ΔR^2=0.008	ΔR^2=0.081**	ΔR^2=0.053**	ΔR^2=0.015	ΔR^2=0.068**	ΔR^2=0.041*	ΔR^2=0.138**	ΔR^2=0.094**	ΔR^2=0.009	ΔR^2=0.099**	ΔR^2=0.022

Note: The alpha values are set to 0.05/10 = 0.005, 0.01/10 = 0.001. **p* < 0.05, with Bonferroni correction. ***p* < 0.01, with Bonferroni correction.

Table S8. Hierarchical regression models predicting academic achievement in Grades 9 of junior high school considering age, gender, cognitive tests, general processing speed（Figure matching）.

Predictors	Mathematics					Chinese					English				
	Step 1	Step 2	Step 3	Step 4	Step 5	Step 1	Step 2	Step 3	Step 4	Step 5	Step 1	Step 2	Step 3	Step 4	Step 5
	β	*β*	*β*	*β*	*β*	*β*	*β*	*β*	*β*	*β*	*β*	*β*	*β*	*β*	*β*
Age(months)	-0.074	-0.049	-0.044	-0.026	-0.018	0.013	0.030	0.030	0.059	0.066	-0.006	0.017	0.018	0.033	0.038
Gender	0.192*	0.173	0.198*	0.158	0.167	0.285**	0.272**	0.279**	0.262**	0.271**	0.371**	0.354**	0.365**	0.322**	0.327**
Nonverbal matrix reasoning		0.340**	0.291**	0.204*	0.197*		0.232**	0.206*	0.136	0.129		0.308**	0.285**	0.197*	0.193*
Mental rotation			0.19*	0.116	0.093			0.084	0.017	-0.004			0.088	0.015	0.002
Spatial working memory			0.023	0.000	-0.014			0.075	0.051	0.039			0.028	0.005	-0.002
Visual tracing				0.184*	0.172				0.230*	0.219*				0.169	0.162
Visual search				0.262**	0.234**				0.138	0.113				0.274**	0.259**
Figure matching					0.110					0.099					0.060
	ΔR^2=0.043	ΔR^2=0.114**	ΔR^2=0.036	ΔR^2=0.098**	ΔR^2=0.009	ΔR^2=0.081**	ΔR^2=0.053**	ΔR^2=0.015	ΔR^2=0.068**	ΔR^2=0.008	ΔR^2=0.138**	ΔR^2=0.094**	ΔR^2=0.009	ΔR^2=0.099**	ΔR^2=0.003

Note: The alpha values are set to 0.05/10 = 0.005, 0.01/10 = 0.001. **p* < 0.05, with Bonferroni correction. ***p* < 0.01, with Bonferroni correction.

Table S9. Hierarchical regression models predicting academic achievement in Grades 9 of junior high school considering age, gender, cognitive tests, specific processing speed（Word semantics）.

Predictors	Mathematics					Chinese					English				
	Step 1	Step 2	Step 3	Step 4	Step 5	Step 1	Step 2	Step 3	Step 4	Step 5	Step 1	Step 2	Step 3	Step 4	Step 5
	β	*β*	*β*	*β*	*β*	*β*	*β*	*β*	*β*	*β*	*β*	*β*	*β*	*β*	*β*
Age(months)	-0.074	-0.049	-0.044	-0.026	-0.024	0.013	0.030	0.030	0.059	0.061	-0.006	0.017	0.018	0.033	0.035
Gender	0.192*	0.173	0.198*	0.158	0.097	0.285**	0.272**	0.279**	0.262**	0.189*	0.371**	0.354**	0.365**	0.322**	0.269**
Nonverbal matrix reasoning		0.340**	0.291**	0.204*	0.168		0.232**	0.206*	0.136	0.093		0.308**	0.285**	0.197*	0.166
Mental rotation			0.19*	0.116	0.105			0.084	0.017	0.004			0.088	0.015	0.006
Spatial working memory			0.023	0.000	-0.001			0.075	0.051	0.051			0.028	0.005	0.005
Visual tracing				0.184*	0.130				0.230*	0.165				0.169	0.121
Visual search				0.262**	0.242**				0.138	0.113				0.274**	0.256**
Word semantics					0.258**					0.312**					0.226**
	ΔR^2=0.043	ΔR^2=0.114**	ΔR^2=0.036	ΔR^2=0.098**	ΔR^2=0.056**	ΔR^2=0.081**	ΔR^2=0.053**	ΔR^2=0.015	ΔR^2=0.068**	ΔR^2=0.081**	ΔR^2=0.138**	ΔR^2=0.094**	ΔR^2=0.009	ΔR^2=0.099**	ΔR^2=0.043**

Note: The alpha values are set to 0.05/10 = 0.005, 0.01/10 = 0.001. *p < 0.05, with Bonferroni correction. **p < 0.01, with Bonferroni correction.

Table S10. Hierarchical regression models predicting academic achievement in Grades 9 of junior high school considering age, gender, cognitive tests, specific processing speed（Simple subtraction）.

Predictors	Mathematics					Chinese					English				
	Step 1	Step 2	Step 3	Step 4	Step 5	Step 1	Step 2	Step 3	Step 4	Step 5	Step 1	Step 2	Step 3	Step 4	Step 5
	β	*β*	*β*	*β*	*β*	*β*	*β*	*β*	*β*	*β*	*β*	*β*	*β*	*β*	*β*
Age(months)	-0.074	-0.049	-0.044	-0.026	-0.026	0.013	0.030	0.030	0.059	0.059	-0.006	0.017	0.018	0.033	0.034
Gender	0.192*	0.173	0.198*	0.158	0.117	0.285**	0.272**	0.279**	0.262**	0.217**	0.371**	0.354**	0.365**	0.322**	0.277**
Nonverbal matrix reasoning		0.340**	0.291**	0.204*	0.167*		0.232**	0.206*	0.136	0.094		0.308**	0.285**	0.197*	0.157
Mental rotation			0.19*	0.116	0.108			0.084	0.017	0.008			0.088	0.015	0.007
Spatial working memory			0.023	0.000	-0.017			0.075	0.051	0.033			0.028	0.005	-0.013
Visual tracing				0.184*	0.165				0.230*	0.209				0.169	0.148
Visual search				0.262**	0.167				0.138	0.031*				0.274**	0.169*
Simple subtraction					0.300**					0.338**					0.332**
	ΔR^2=0.043	ΔR^2=0.114**	ΔR^2=0.036	ΔR^2=0.098**	ΔR^2=0.072**	ΔR^2=0.081**	ΔR^2=0.053**	ΔR^2=0.015	ΔR^2=0.068**	ΔR^2=0.091**	ΔR^2=0.138**	ΔR^2=0.094**	ΔR^2=0.009	ΔR^2=0.099**	ΔR^2=0.088**

Note: The alpha values are set to 0.05/10 = 0.005, 0.01/10 = 0.001. *$p < 0.05$, with Bonferroni correction. **$p < 0.01$, with Bonferroni correction.

Table S11. Hierarchical regression models predicting academic achievement in Grades 9 of junior high school considering age, gender, cognitive tests, specific processing speed（Complex subtraction）.

Predictors	Mathematics					Chinese					English				
	Step 1	Step 2	Step 3	Step 4	Step 5	Step 1	Step 2	Step 3	Step 4	Step 5	Step 1	Step 2	Step 3	Step 4	Step 5
	β	*β*	*β*	*β*	*β*	*β*	*β*	*β*	*β*	*β*	*β*	*β*	*β*	*β*	*β*
Age(months)	-0.074	-0.049	-0.044	-0.026	-0.017	0.013	0.030	0.030	0.059	0.069	-0.006	0.017	0.018	0.033	0.044
Gender	0.192*	0.173	0.198*	0.158	0.134	0.285**	0.272**	0.279**	0.262**	0.237**	0.371**	0.354**	0.365**	0.322**	0.295**
Nonverbal matrix reasoning		0.340**	0.291**	0.204*	0.188*		0.232**	0.206*	0.136	0.119		0.308**	0.285**	0.197*	0.179*
Mental rotation			0.19*	0.116	0.119			0.084	0.017	0.021			0.088	0.015	0.019
Spatial working memory			0.023	0.000	-0.007			0.075	0.051	0.044			0.028	0.005	-0.003
Visual tracing				0.184*	0.171				0.230*	0.216				0.169	0.154
Visual search				0.262**	0.162				0.138	0.033				0.274**	0.160
Complex subtraction					0.254**					0.267**					0.292**
	$\Delta R^2=0.043$	$\Delta R^2=0.114$**	$\Delta R^2=0.036$	$\Delta R^2=0.098$**	$\Delta R^2=0.051$**	$\Delta R^2=0.081$**	$\Delta R^2=0.053$**	$\Delta R^2=0.015$	$\Delta R^2=0.068$**	$\Delta R^2=0.057$**	$\Delta R^2=0.138$**	$\Delta R^2=0.094$**	$\Delta R^2=0.009$	$\Delta R^2=0.099$**	$\Delta R^2=0.068$**

Note: The alpha values are set to 0.05/10 = 0.005, 0.01/10 = 0.001. *$p < 0.05$, with Bonferroni correction. **$p < 0.01$, with Bonferroni correction.

Table S12. Hierarchical regression models predicting academic achievement in Grades 9 of junior high school considering age, gender, cognitive tests、specific processing speed（Complex multiplication）.

Predictors	Mathematics					Chinese					English				
	Step 1	Step 2	Step 3	Step 4	Step 5	Step 1	Step 2	Step 3	Step 4	Step 5	Step 1	Step 2	Step 3	Step 4	Step 5
	β	*β*	*β*	*β*	*β*	*β*	*β*	*β*	*β*	*β*	*β*	*β*	*β*	*β*	*β*
Age(months)	-0.074	-0.049	-0.044	-0.026	-0.033	0.013	0.030	0.030	0.059	0.050	-0.006	0.017	0.018	0.033	0.025
Gender	0.192*	0.173	0.198*	0.158	0.151	0.285**	0.272**	0.279**	0.262**	0.253**	0.371**	0.354**	0.365**	0.322**	0.313**
Nonverbal matrix reasoning		0.340**	0.291**	0.204*	0.193*		0.232**	0.206*	0.136	0.122		0.308**	0.285**	0.197*	0.184
Mental rotation			0.19*	0.116	0.129			0.084	0.017	0.034			0.088	0.015	0.032
Spatial working memory			0.023	0.000	-0.067			0.075	0.051	-0.036			0.028	0.005	-0.081
Visual tracing				0.184*	0.165				0.230*	0.206*				0.169	0.145
Visual search				0.262**	0.231**				0.138	0.097				0.274**	0.234**
Complex multiplication					0.164					0.214*					0.211*
	ΔR^2=0.043	ΔR^2=0.114**	ΔR^2=0.036	ΔR^2=0.098**	ΔR^2=0.020	ΔR^2=0.081**	ΔR^2=0.053**	ΔR^2=0.015	ΔR^2=0.068**	ΔR^2=0.034*	ΔR^2=0.138**	ΔR^2=0.094**	ΔR^2=0.009	ΔR^2=0.099**	ΔR^2=0.033*

Note: The alpha values are set to 0.05/10 = 0.005, 0.01/10 = 0.001. *p < 0.05, with Bonferroni correction. **p < 0.01, with Bonferroni correction.
